# Interplay between Endoplasmic Reticulum Stress and Large Extracellular Vesicles (Microparticles) in Endothelial Cell Dysfunction

**DOI:** 10.3390/biomedicines8100409

**Published:** 2020-10-12

**Authors:** Aisha Osman, Tarek Benameur, Hesham M. Korashy, Asad Zeidan, Abdelali Agouni

**Affiliations:** 1 Department of Pharmaceutical Sciences, College of Pharmacy, QU health, Qatar University, Doha 2713, Qatar; am1002749@student.qu.edu.qa (A.O.); hkorashy@qu.edu.qa (H.M.K.); 2 Department of Biomedical Sciences, College of Medicine, King Faisal University, P.O. Box 400, Al Ahsa 31982, Saudi Arabia; tbenameur@kfu.edu.sa; 3 Department of Basic Medical Sciences, College of Medicine, QU health, Qatar University, Doha 2713, Qatar; a.zeidan@qu.edu.qa

**Keywords:** extracellular vesicles (EVs), microparticles (MPs), endoplasmic reticulum (ER) stress, endothelial dysfunction, cardiovascular disease, diabetes, metabolic syndrome

## Abstract

Upon increased demand for protein synthesis, accumulation of misfolded and/or unfolded proteins within the endoplasmic reticulum (ER), a pro-survival response is activated termed unfolded protein response (UPR), aiming at restoring the proper function of the ER. Prolonged activation of the UPR leads, however, to ER stress, a cellular state that contributes to the pathogenesis of various chronic diseases including obesity and diabetes. ER stress response by itself can result in endothelial dysfunction, a hallmark of cardiovascular disease, through various cellular mechanisms including apoptosis, insulin resistance, inflammation and oxidative stress. Extracellular vesicles (EVs), particularly large EVs (lEVs) commonly referred to as microparticles (MPs), are membrane vesicles. They are considered as a fingerprint of their originating cells, carrying a variety of molecular components of their parent cells. lEVs are emerging as major contributors to endothelial cell dysfunction in various metabolic disease conditions. However, the mechanisms underpinning the role of lEVs in endothelial dysfunction are not fully elucidated. Recently, ER stress emerged as a bridging molecular link between lEVs and endothelial cell dysfunction. Therefore, in the current review, we summarized the roles of lEVs and ER stress in endothelial dysfunction and discussed the molecular crosstalk and relationship between ER stress and lEVs in endothelial dysfunction.

## 1. Introduction

Extracellular vesicles (EVs) are liberated from cells upon cellular activation or stress. EVs are typically classified based on their size and mechanism of formation and release from their parent cells. The main EVs subtypes are exosomes, apoptotic bodies and large size EVs (lEVs) also commonly referred to as microparticles (MPs) [1,2]. The content and circulating levels of lEVs or MPs change dynamically in response to specific external signals. They could carry cargo content including secretable and non-secretable biological molecules including active lipids, membrane and cytosolic proteins, nucleic acids such as coding (mRNAs) and noncoding RNAs (e.g., microRNAs and long noncoding RNAs) and subcellular organelles from the parent cell and transfer them to target cells [3]. Therefore, lEVs are nowadays recognized as true vectors of intercellular communication and mediators of many biological messages and provide a snapshot of the cell of origin under physiological and pathological conditions.

Endoplasmic reticulum (ER) is a major site for many cellular functions including post-translational folding and synthesis of secretory and transmembrane proteins in eukaryotic cells. Under various physiological or pathophysiological conditions that increase protein demand or result in the accumulation of misfolded and/or unfolded proteins within the ER lumen, a state of ER stress takes place. To attenuate protein synthesis, an adaptive signaling cascade called unfolded protein response (UPR) is activated, which aims at increasing protein folding capacity and promoting degradation of irreversibly misfolded proteins in an attempt to restore ER homeostasis [4]. However, the chronic activation of UPR leads to a state of ER stress and the activation of pro-apoptotic signals [5]. Recent findings have highlighted the significance of ER homeostasis in endothelial cell function. In spite of the helpful actions of the transient activation of UPR, the pathologic chronic activation ER stress leads to endothelial cell dysfunction and subsequently to apoptosis and inflammation contributing thus to progression of cardiovascular disease [5,6,7].

Given the complexity of endothelial function, various studied biomarkers were not able to provide consistent information about the activation state of endothelium when investigated alone. In this context, lEVs (MPs) were explored as circulating biomarkers under various pathological conditions associated with endothelial dysfunction and showed great promise [8,9]. Furthermore, the role of lEVs was also examined in the pathogenesis of endothelial dysfunction and several underlying mechanisms have been tested [10]. Recently, the role of ER stress in endothelial dysfunction induced by lEVs (MPs) was highlighted [11]. It has been reported that treatment of human aortic endothelial cells in vitro with lEVs (MPs) derived from patients with metabolic syndrome or generated from apoptotic T-lymphocytes, were able to activate all UPR pathways, reduced nitric oxide (NO) bioavailability and impaired endothelium-dependent vasorelaxation in mice. Moreover, the pharmacological inhibition of ER stress abrogated the effects mediated by lEVs [11]. Furthermore, lEVs generated from endothelial cells subjected to ER stress were able to cause ER stress in a vicious circle and mediate endothelial cell dysfunction in naïve cells [12]. These studies highlight the importance of ER stress as a novel bridging molecular link between EVs and endothelial cell dysfunction, the initiating step in cardiovascular disease.

In the current review, we summarized the roles of lEVs and ER stress in endothelial cell dysfunction and discussed the crosstalk between them in mediating cell dysfunction. Better understanding the molecular origins behind endothelial cell dysfunction will support identifying novel therapeutic targets to reduce the burden of cardiovascular disease on mortality and morbidity.

## 2. Physiology of Endothelium and Endothelial Cell Dysfunction

The endothelium is the inner lining of blood vessels that separates the blood from the vessel wall [13]. The endothelium regulates vascular tone, vascular homeostasis and angiogenesis by producing and secreting relaxing and contracting factors, prothrombotic and antithrombotic factors, growth factors and inflammatory mediators. Table 1 summarizes the main factors produced and released by the endothelium.

Among all factors produced by the endothelium, nitric oxide (NO) is the main vasorelaxant synthesized by endothelial cells by converting l-arginine to l-citrulline and NO by the endothelial NO synthase enzyme (eNOS) [14]. NO contributes to maintaining vascular tone and health through diffusing to smooth muscle cells and activating the enzyme soluble guanylate cyclase. This enzyme is required for the production of cyclic guanosine monophosphate (cGMP), which activates cGMP-dependent kinase, which in turn phosphorylates K^+^ channels resulting in hyperpolarization and decrease in intracellular Ca^2+^ level [15]. Low intracellular Ca^2+^ level leads to vascular smooth muscle cells relaxation, vascular smooth muscle cells are located under the endothelium and are directly affected by the state of endothelium. In addition to the role of NO in maintaining vascular tone, it also maintains vascular hemostasis by inhibiting platelet adhesion and aggregation to the endothelium and preventing the production of adhesion molecules [16], all of which are protective effects against atherosclerosis triggered by prothrombotic and proinflammatory conditions [17]. Additionally, NO inhibits the proliferation and migration of smooth muscle cells [18,19].

The endothelium also has a role in angiogenesis—the formation of new blood vessels from pre-existing ones. The endothelium releases growth factors that are important for cell proliferation and migration, the two steps required for forming new blood vessels. Angiogenesis is important for growth, reproduction and wound healing. However, in many disease conditions, angiogenesis is perturbed with impaired angiogenesis observed in diabetes, diabetic neuropathy and ischemic tissue injuries, while excessive angiogenesis response was observed in cancer development, diabetic retinopathy and diabetic nephropathy [20].

In vascular homeostasis, there is a balance between relaxing and contracting factors. Additionally, a balance between antithrombotic and prothrombotic factors is important for the normal function of the vascular system. Perturbation of endothelial functions in diseases such as diabetes and insulin resistance states disturb this balance leading to what is known as “endothelial dysfunction”. Endothelial dysfunction is the early step in atherogenesis, which results in cardiovascular complications in patients with cardiovascular risk factors such as obesity, diabetes and metabolic syndrome [21]. Features of endothelial dysfunction in diabetes include:

- Impairment of vascular function indicated by reduced NO bioavailability, impaired endothelium-depended smooth muscle relaxation and perturbated angiogenesis;
- Induction of vascular inflammation marked by increased production of inflammatory mediators and adhesion molecules;
- Activation of a prothrombotic state by increasing production of procoagulant factors and enhancing platelet aggregation.

Increased demand for protein synthesis in diabetes and insulin resistance states increases the protein folding load on the ER resulting in disruption of the ER balance and activation of ER stress response [22]. ER stress response activation contributes to the onset of endothelial dysfunction at the molecular level [5,7].

## 3. Endoplasmic Reticulum (ER) Stress

**Figure 1:**
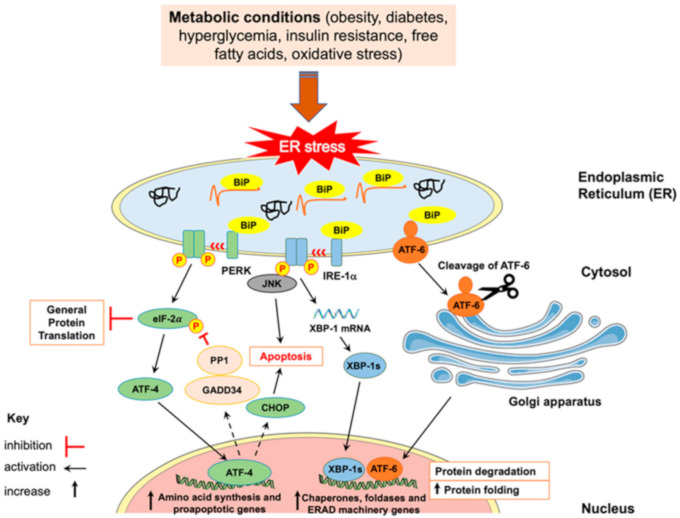
Cellular pathways involved in unfolded protein response (UPR) and endoplasmic reticulum (ER) stress response activation. At resting state, the UPR stress sensors, protein kinase R (PKR)-like ER kinase (PERK), inositol-requiring enzyme (IRE-1α) and activating transcription factor (ATF-6) are kept in place by the chaperone immunoglobulin heavy chain binding protein (BiP). During metabolic stress conditions, such as in obesity and diabetes, accumulation of unfolded/misfolded proteins in the ER lumen leads to dissociation of BiP from UPR stress sensors to fix the unfolded/misfolded proteins inside the ER, while the stress sensors of UPR become available to activate downstream signaling. (i) PERK autophophorylates and dimerizes to become active and activates eukaryotic initiation factor (eIF-2α) through phosphorylation. Active eIF-2α attenuates general translation of proteins but selectively enhances the translation of ATF-4, which is then moved to the nucleus to promote transcription of genes involved in amino acid synthesis and apoptosis. ATF-4 is also involved in the negative feedback of PERK signaling by enhancing transcription of growth arrest and DNA damage inducible protein (GADD34) that activates protein phosphatase 1 (PP1) leading to PERK dephosphorylation and hence inactivation; (ii) IRE-1α autophosphorylates and dimerizes to be activated, then it splices X box-binding protein 1 (XBP-1) mRNA to the active XBP-1s. XBP-1s then translocates into the nucleus; (iii) ATF-6 is cleaved by proteases in the Golgi apparatus to become active. Both XBP-1s and cleaved ATF-6 move to the nucleus and facilitate the transcription of genes involved in protein folding and degradation (ER-associated degradation (ERAD) machinery). c-Jun-NH2-terminal kinase (JNK) proinflammatory and proapoptotic pathway is closely related to IRE-1α and can be activated after prolonged ER stress response [4,5].

The ER is a cell organelle that performs many cellular functions including post-translational folding and synthesis of secretory and transmembrane proteins. Upon various physiological or pathological disturbances that increase protein demand or lead to the accumulation of misfolded or unfolded proteins within the ER lumen, an adaptive signaling cascade called UPR is activated. UPR is activated to attenuate protein synthesis, increase protein folding capacity and promote degradation of irreversibly misfolded proteins in an attempt to restore ER homeostasis [4]. If ER homeostasis is not restored, ER stress response is activated, which triggers signaling pathways involved in cell apoptosis. Figure 1 represents the activation of UPR and ER stress response and the involved cellular pathways.

Three transmembrane sensors located on the ER surface: protein kinase R (PKR)-like ER kinase (PERK), inositol-requiring enzyme (IRE)-1α and activating transcription factor (ATF)-6, are activated following their dissociation from the major ER chaperone: immunoglobulin heavy chain binding protein (BiP) also known as glucose-regulated protein (GRP)-78. The ER chaperone, BiP, becomes available in the ER lumen to bind to and correct the misfolded and unfolded polypeptide chains, while the activated transmembrane sensors are involved in UPR downstream signaling pathways (for review see [4,6,23]) as follows:

a. After its dissociation from BiP, PERK undergoes dimerization and autophosphorylation, then it phosphorylates eukaryotic initiation factor (eIF)-2α at the alpha subunit. Phosphorylated eIF-2α reduces the general translation of mRNA in order to alleviate the load on the ER; however, it allows, selectively, the translation of ATF-4 mRNA. ATF-4, as a transcription factor, stimulates the expression of genes encoding proteins involved in amino acid biosynthesis and cell death such as tribbles homolog 3 (TRIB3) and CCAAT/enhancer binding proteins (C/EBP) homologous protein (CHOP), respectively. ATF-4 also controls the negative feedback of PERK activation by enhancing the expression of growth arrest and DNA damage inducible protein (GADD34), which leads to the dephosphorylation of eIF-2α by protein phosphatase 1 (PP1).
b. IRE-1α endoribonuclease activity is mediated by its dimerization and autophosphorylation. Activated IRE-1α splices X box-binding protein 1 (XBP-1) mRNA to form active spliced form (XBP-1s), which then translocates into the nucleus and promotes the transcription of genes encoding ER-associated degradation (ERAD) proteins and ER chaperones. Upon prolonged ER stress, IRE-1α can activate inflammatory and apoptotic pathways. For example, IRE-1α can interact with tumor necrosis factor (TNF) receptor-associated factor (TRAF)-2 to promote activation of nuclear factor κB (NF-κB), which is involved in the upregulation of inflammatory genes. Additionally, IRE-1α/TRAF-2 can activate c-Jun-NH2-terminal kinase (JNK), which was shown to induce insulin resistance, inflammation and apoptosis. Additionally, TRAF-2 stimulates apoptosis-signaling-kinase 1 (ASK-1), which can enhance the activity of p38 mitogen activated protein kinase (MAPK), which can result in the activation of proapoptotic CHOP.
c. After release from BiP, ATF-6 translocates into the Golgi apparatus to be cleaved by proteases, site-1 protease (S1P) and site-2 protease (S2P). Then, the transcriptionally active cytosolic domain of ATF-6 is released and translocates into the nucleus to trigger the transcription of genes encoding for proteins involved in degradation of irreversibly misfolded proteins (ERAD machinery proteins) and folding of proteins (ER chaperones such as BiP and GRP94).

Collectively, UPR is a defensive mechanism activated to restore the proper function of the ER. However, upon its prolonged activation, a state of ER stress takes place. As described below, ER stress response by itself can result in endothelial dysfunction through various underlying molecular mechanisms including apoptosis, insulin resistance, inflammation, autophagy and oxidative stress.

### 3.1. ER Stress-Mediated Inflammation and Apoptosis

The transmembrane stress sensor located in the ER lumen, IRE-1α, can interact with TRAF-2 to activate JNK pathway [24], leading to downstream activation of inflammatory signaling and cell apoptosis [25]. Moreover, ATF-4 that is selectively activated by eIF-2α during ER stress translocates into the nucleus and facilitates the transcription of proapoptotic factor CHOP, which leads to ER stress-mediated apoptosis. Since eIF-2α is activated to attenuate general translation of proteins with the aim to decrease protein load on the ER, it also inhibits the translation of IκB, the inhibitor of NF-κB, leading to increased activity of NF-κB [26,27]. NF-κB can move to the nucleus leading to the transcription of proinflammatory factors and inflammation. Additionally, upon cleavage of ATF-6 and its translocation into the nucleus, it can promote the transcription of CHOP and NF-κB as well as other genes involved in protein folding and degradation [28]. Altogether, UPR arms relate to apoptosis, inflammation and oxidative stress, all of which are underlying mechanisms associated with prolonged ER stress and can explain the link between ER stress and endothelial dysfunction seen in metabolic conditions.

### 3.2. ER Stress-Mediated Oxidative Stress

ER stress is connected with oxidative stress in a vicious circle [5]. Increased demand for protein synthesis in the cell leads to increased formation of non-native disulfide bonds. This triggers the antioxidant glutathione to scavenge the formed reactive oxygen species (ROS). With time, glutathione stores deplete resulting in oxidative stress; oxidative stress is the state of imbalance between ROS generation and antioxidant protection. Moreover, folding of proteins requires adenosine triphosphate (ATP) and results in ROS generation. Accumulation of misfolded proteins lead to increased demand for ATP synthesis and further production of ROS. Additionally, the crosstalk between the two cellular organelles mitochondria and ER during the UPR response stimulates ATP production through transferring large amount of Ca^2+^ from ER to mitochondria, which further enhances ROS production by the mitochondria. Reduced levels of Ca^2+^ in the ER affects the function of Ca^2+^-dependent chaperones and stimulates more ER stress. The antioxidant heme oxygenase (HO)-1 enzyme was found to protect against ER stress-mediated oxidative stress in endothelial cells, in addition to reversing endothelial dysfunction [29]. Moreover, ER stress was shown to reduce the expression of superoxide dismutase (SOD) in mouse adipocytes [30]. The decrease in the activity of SOD is also connected with endothelial dysfunction [31].

### 3.3. ER Stress-Mediated Autophagy

ER stress is closely linked to apoptosis, which can result after sustained ER stress as we discussed. Autophagy, which is important for the clearance of toxic proteins and recycling of cytosolic content, was found to be activated as a protective mechanism to decrease unfolded proteins load and prevent ER-stress mediated apoptosis [32]. Autophagy begins with the formation of isolation membrane where microtubule-associated protein light chain (LC3)-II, the phosphatidylethanolamine conjugated with LC3-I, binds to allow elongation of the isolation membrane and formation of autophagosome. Autophagosome is a membranous vesicle that sequesters cytoplasmic components and organelles that need to be degraded. Autophagosome membrane then fuses with lysosomal membrane to form autolysosome where the content is degraded, and macromolecules are recycled and delivered to the cell again. Autophagy has been linked to the three arms of UPR [33]. For example, active ATF-4 enhances the transcription of autophagy genes: microtubule-associated protein 1 light chain 3 (*MAP1LC3B*), Beclin-1 (*BECN1*), autophagy-related 3 (*ATG3*) and autophagy-related 12 (*ATG12*). Additionally, cleaved ATF-6 increases the expression of death-associated protein kinase 1 (DAPK1). DAPK1 phosphorylates BECN1 leading to autophagy. The activation of IRE-1α results in the activation of MAPK leading to the end of the activation of autophagy [34].

### 3.4. ER Stress-Mediated Insulin Resistance

ER stress is activated in conditions characterized by insulin resistance such as obesity and diabetes [35]. At the molecular level, ER stress contributes to insulin resistance via inhibition of insulin signaling [5]. Inhibition of insulin signaling is mediated by both IRE-1α and PERK arms of the UPR pathway. IRE-1α activates JNK, which results in inhibition of insulin receptor substrate (IRS)-1. While PERK activates eIF-2α, which attenuates protein translation including the translation of protein kinase B (PKB or Akt), PKB pathway is one of the major pathways that mediate insulin signaling. Moreover, eIF-2α selectively enhances the translation of ATF-4, which, in turn, activates the transcription of tribbles homolog 3 (*TRIB3*) and leads to synthesis of PKB inhibitory kinase. PKB inhibitory kinase inhibits PKB terminating this insulin signaling pathway. Additionally, the accumulation of misfolded proinsulin in the ER lumen prevents insulin from releasing and subsequently impairs insulin signaling. Insulin resistance is a major contributor to endothelial dysfunction since insulin plays key vascular functions. Endothelial cells express insulin receptor at their surface and its activation stimulates PKB, which can directly stimulate the activity of eNOS causing an increased release of NO and subsequent relaxation of adjacent smooth muscle cells [36]. Insulin resistance disturbs the functions of the endothelium [37]. Okon et al. [38] observed that phosphorylation of PKB was reduced by half in vessels obtained from diabetic patients compared to controls and that this reduction was associated with impaired endothelium-dependent vasodilation [38]. Furthermore, it has been observed that genetically modified mice overexpressing a defective mutant of insulin receptor, specifically in endothelial cells, had a dramatic decrease in NO production and impaired endothelium-dependent vasorelaxation [39].

## 4. ER Stress and Endothelial Dysfunction

ER stress was shown to be involved in endothelial dysfunction, which is known to occur in diabetes and insulin resistance states [6]. Moreover, ER stress is linked to atherosclerosis as it was found to be activated in all stages of atherosclerosis [40]. Atherosclerosis is a consequence of endothelial dysfunction, which can progress to microvascular and macrovascular complications in diabetes. Several human, in vivo and in vitro studies investigated the link between ER stress and endothelial dysfunction and found that endothelial dysfunction can happen through ER stress response activation [5].

In obesity, for example, ER stress was found to be activated, and was associated with fetoplacental endothelial dysfunction at birth in obese pregnant women [41]. In addition, plasma collected from obese children reduced insulin-stimulated NO production, a feature of endothelial cell dysfunction, in human umbilical vein endothelial cells (HUVECs), with concomitant ER stress activation [42]. Administration of ER stress inhibitors, 4-phenylbutyric acid (PBA) or tauroursodeoxycholic acid (TUDCA), reversed endothelial dysfunction in HUVECs indicating that impaired NO production happened through ER stress activation. In obese adults with established endothelial dysfunction, quantitative immunofluorescence in antecubital veins showed that all UPR stress arms, PERK, IRE-1α and ATF-6, were activated [43]. These findings underscore the role of ER stress in endothelial dysfunction observed during obesity and insulin resistance states.

Diabetes is also associated with endothelial dysfunction; however, the underpinning mechanisms are not fully elucidated. ER stress was investigated recently to determine its role in high glucose-induced endothelial dysfunction. In vitro, HUVECs stimulated with intermittent high glucose for five consecutive days activated ER stress response evident by the upregulation of BiP, ATF-4 and phosphorylated eIF-2α proteins, in addition to overexpression of *BiP*, *CHOP* and *ATF-4* genes, in comparison to controls. Furthermore, it induced endothelial dysfunction by reducing NO bioavailability, causing cell death and impairing angiogenesis. All these effects were reversed after treatment with the ER stress chemical chaperone, PBA, indicating the involvement of ER stress in endothelial dysfunction is induced by high glucose in vitro [29]. Bhatta et al. [44] assessed the activation of ER stress in bone marrow-derived early outgrowth cells (EOCs) grown in high glucose conditions and in EOCs collected from *db*/*db* diabetic mice. They reported dramatic increase in the expression of ER stress markers compared to control conditions and animals. Additionally, angiogenic capacity was found to be reduced in EOCs exposed to high glucose, which exhibited reduced capacity to form colonies, higher apoptosis and impaired migrating capacity, these effects were prevented in the presence of chemical chaperone PBA [44]. Similar results were also shown in vivo. For example, Suganya et al. [45] found an increased immunoreactivity to CHOP and endothelin-1, a strong vasoconstricting agent used as a marker of endothelial dysfunction, and a decreased expression of vascular endothelial growth factor (VEGF) and its receptor (VEGFR2), a key proangiogenic factor, in the pancreatic tissues of streptozotocin (STZ)-induced diabetic rats. When CHOP pathway was inhibited by quercetin, a natural flavonoid known to reduce ER stress, in HUVECs, endothelial function was restored [45]. Similar to in vitro studies, prolonged administration of PBA attenuated endothelial dysfunction in STZ-induced diabetic rats [46]. Choi et al. [47] showed also that *db*/*db* type-2 diabetic mice had higher protein expression of ER stress markers: BiP, phosphorylated IRE-1α and its downstream XBP-1, phosphorylated PERK and its downstream phosphorylated elF-2α, compared to control animals [47]. Furthermore, mouse coronary arteries had impaired endothelium-dependent vasorelaxation and a significant increase in intercellular adhesion molecule (ICAM)-1 and vascular cell adhesion molecule (VCAM)-1, endothelial dysfunction was reversed by the administration of a chemical chaperone, TUDCA [47].

**Figure 2:**
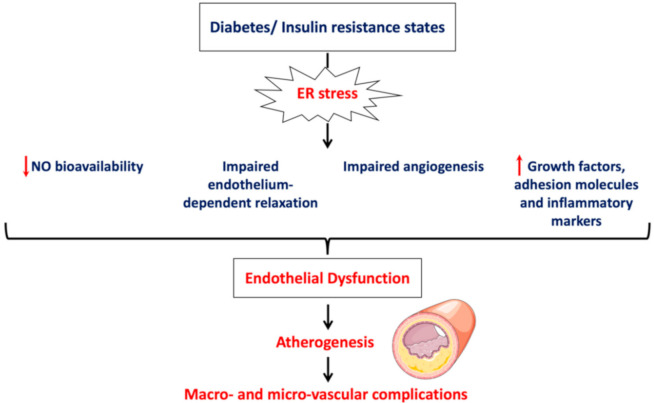
Characteristic features of endothelial dysfunction induced by diabetes through ER stress. In diabetes and insulin resistance states, ER stress was shown to be activated and mediate, in part, endothelial dysfunction, which is the first step in atherosclerosis leading to cardiovascular complications.

Therefore, ER stress is a key underlying mechanism of endothelial dysfunction associated with cardiovascular risk factors and insulin resistance conditions such as obesity and diabetes. Endothelial dysfunction by itself is the first step in the atherogenesis process, which progresses to microvascular and macrovascular complications of diabetes. Figure 2 summarizes the features of endothelial cell dysfunction induced by ER stress in diabetes.

A wide range of drugs and strategies were shown to improve ER stress in cardiovascular conditions. Of note, low molecular weight chemical chaperones, PBA and TUDCA, which are both approved by the US Food and Drug Administration (FDA), were shown to improve protein folding and reduce ER stress response both in cultured cells and in in vivo studies. These compounds were reported to improve glucose response and insulin signaling, endothelial cell dysfunction, hypertrophy, fibrosis and atherosclerosis [48,49].

Multiple regulators and modulators for the various arms of the UPR were reported in the literature [48,49]. Several agents were found to enhance the expression levels of BiP in animal models. For example, valproate, an anti-epileptic drug, has been shown to prevent cell death of retinal epithelial cells in mice subjected to ischemia/reperfusion by increasing BiP and reducing CHOP expression levels [50]. Several FDA-approved drugs used for the treatment of hypertension were also found to alleviate ER stress, such as the angiotensin II receptor blocker, telmisartan, which was found to inhibit the apoptotic signaling response downstream to IRE-1α [51]. The exposure of diabetic rats to olmesartan, another angiotensin II receptor blocker, improved ER stress-mediated renal cell death in rat kidneys [52]. Anti-hypertensive drug, enalapril, an inhibitor of the angiotensin converting enzyme (ACE), was also found to protect against ER stress-mediated hypertension caused by high methionine diet [53].

Some hormonal drugs were also found to alleviate ER stress response. Ghrelin, a hormonal peptide secreted by the stomach, was observed to reduce atherosclerotic plaque in apolipoprotein E-deficient (ApoE^−/−^) mouse model of atherosclerosis by inhibiting ER stress response in endothelial cells [54]. Direct inhibition of UPR effectors by novel small molecules was also reported. A potent and selective competitive inhibitor of IRE-1α, kinase inhibiting RNase attenuator 6 (KIRA6), was found to block the ability of IRE-1α to dimerize and hence protected cells from ER stress-mediated apoptosis. In addition, the treatment with KIRA6 improved cell death of pancreatic β-cells in Akita diabetic mice [55]. Multiple natural compounds were also reported to improve ER stress response [48,49]. Oleanolic acid, for example, improved ER stress and ROS production in diabetic rats [56]. The antioxidant flavonoid, quercetin, prevented the deleterious effects of pharmacologically (thapsigargin)-induced ER stress in LS180 cells, by reducing BiP protein expression and inhibiting the actions of IRE-1α and PERK [57].

## 5. Extracellular Vesicles (EVs)

The International Society for Extracellular Vesicles (ISEV) has endorsed EVs as a general term for the population of vesicles released from the cells, surrounded by a lipid bilayer with no replication capability-like cells [58]. EVs are typically classified in the literature according to their diameter size and mechanism of biogenesis and release from their originating cells. The main EVs subtypes are exosomes, apoptotic bodies and large size EVs (lEVs), also commonly known as microparticles (MPs). Exosomes range in size from 40–120 nm and are released from cells by fusion of multivesicular bodies (MVBs) with cell membrane and then MVBs release exosomes to the extracellular environment by exocytosis [1,59]. While apoptotic bodies (size >1000 nm) and lEVs (MPs) (size 100–1000 nm) appear to have similar mechanism of release, which is outward blebbing and shedding of vesicles directly from the plasma membrane of apoptotic cells or activated cells, respectively [1,2].

Due to the differences in the physical characteristics of EVs, there are different methods for isolating and studying them. The most commonly used method for isolating EVs is deferential centrifugation (DC) [60]. DC allows the separation of EV subtypes by applying different centrifugation speeds. Increasing the centrifugation speed will pellet the smaller size EVs. For example, lEVs (MPs) are isolated by serial centrifugations at ~21,000 gravitational force (× *g*) while exosomes are isolated at ~100,000× *g*. Before EV isolation, cells and cell debris are removed by centrifugation at 200–1500× *g* for 5–10 min and 2000× *g* for 10–30 min, respectively, as indicated in most protocols of EVs isolation [3]. It is important to note that methods of isolation only enable the enrichment with a specific EV population but not actual separation.

In addition, protein concentration assay, tunable resistive pulse sensing (TRPS), nanoparticle tracking analysis (NTA), flow cytometry and transmission electron microscopy (TEM) are methods used for studying EVs. Protein assay is an indirect method of quantifying EVs by measuring the total protein content of EVs. The limit of this method is the inaccuracy due to contamination with copurified proteins with EVs. TRPS and NTA allow the detection of single EVs with minimal detectable size of 70–100 nm and 70–90 nm, respectively [61]. However, these two methods are laborious and time consuming, which limit their use in studying clinical samples. On the other hand, flow cytometry is widely used for detecting and quantifying EVs with different sizes and from different origins, subtypes of EVs are enriched with specific proteins that can be used as markers for their detection by flow cytometry. Although the minimal detectable vesicle size by flow cytometry is 150–190 nm, which means that it cannot detect EVs of less than 120 nm (exosomes), it is the widely preferred method due to its high speed. In addition to the detection limit of flow cytometry, there are great differences between flow cytometers in relation to the optical configurations and the light scattering between EVs and the reference material used. Therefore, an initiative was taken by the Scientific Standardization Committee on Vascular Biology of the International Society on Thrombosis and Hemostasis (ISTH) in 2014 to standardize the measurements of EVs among laboratories using flow cytometry, which is a crucial step for generating comparable data. In addition, advances in technology will allow the use of highly sensitive flow cytometers. Lastly, transmission electron microscopy can be used for imaging all types of EVs [1].

### 5.1. Mechanisms of lEVs (MPs) Formation

**Figure 3:**
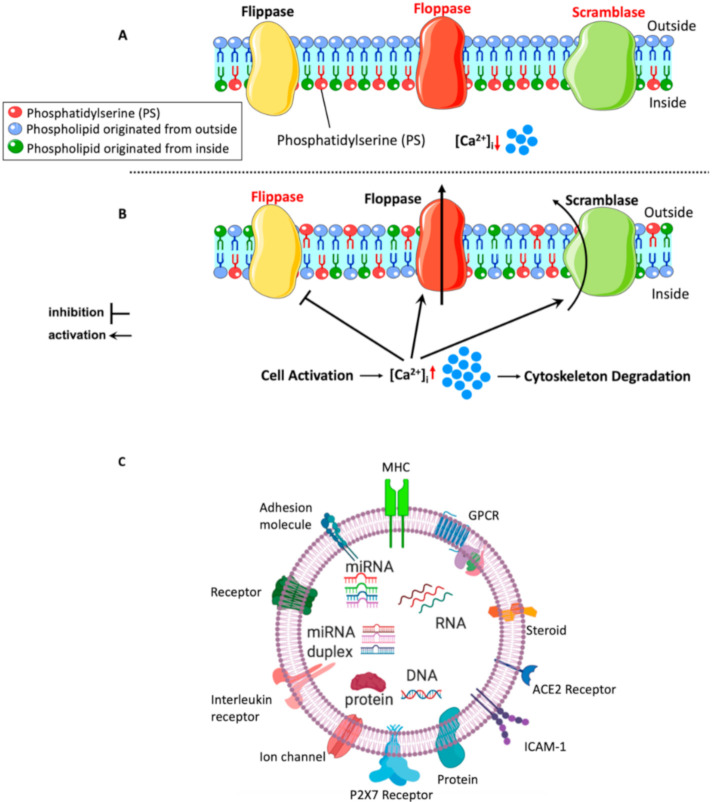
Mechanism of large size extracellular vesicles (lEV) (microparticles: MP) formation (**A**,**B**) and cargo content of lEVs (MPs) in biological materials and cell surface proteins and antigens (**C**). (**A**) At resting state, Ca^+2^ levels are low and membrane lipid distribution is maintained by flippase enzyme with phosphatidylserine kept in the inner leaflet of the plasma membrane; (**B**) upon cell activation, Ca^+2^ levels are elevated leading to disruption of the cytoskeleton proteins. Moreover, Ca^+2^ inhibits the action of flippase enzyme and activates floppase and scramblase enzymes. Floppase translocates inner leaflet lipids to the outer leaflet and scramblase moves lipids down their concentration gradient, leading to externalization of phosphatidylserine and membrane remodeling. These mechanisms are thought to be involved in lEV (MP) formation. Abbreviations: lEVs, large extracellular vesicles; MPs, microparticles; MHC, major histocompatibility complex; GPCR, G-protein coupled receptor; ICAM-1, intercellular adhesion molecule-1.

Amongst these EVs, lEVs (MPs) were one of the most studied in physiology and pathophysiology. They are released from activated cells by outward blebbing of the plasma membrane as mentioned earlier, but the exact molecular mechanisms underlying lEV biogenesis are not known. However, studies have indicated that lEV formation begins after the activation of the cell by an agonist or shear stress, or when the cell is undergoing apoptosis, and subsequent elevation in Ca^2+^ levels. The Ca^2+^ surge results in disruption of the cytoskeleton proteins, and plasma membrane remodeling due to loss of membrane asymmetry and the externalization of negatively charged phosphatidylserine (PS) to the outer leaflet of the membrane [62]. Figure 3A,B summarize the mechanisms of lEV formation.

Disruption of the cytoskeleton, which supports the plasma membrane, is one of the important events that are believed to be involved in lEV formation. For example, inhibition of actin polymerization, which is important for cell membrane structure and motility, resulted in increased lEV shedding from platelets [63] and megakaryocytes [64]. Moreover, calpain, which is a Ca^2+^-dependent protease that cleaves cytoskeletal proteins such as α-actin, when inhibited, was shown to decrease formation of lEVs derived from platelets [65] and neutrophils [66] in vitro. Nonetheless, calpain activity was not altered after induction of endothelial cells with angiotensin II to produce endothelial lEVs [67]. This suggests that the cellular origin of MPs might determine the activity of calpain during lEV formation. Other regulators of the cytoskeletal structure and dynamics are Rho kinase [67] and transglutaminase 2, they have been shown to have a role in lEV formation in endothelial and vascular smooth muscle cells, respectively [68].

Externalization of PS, an aminophospholipid located in the inner leaflet of the plasma membrane [69], is also a key event that happens during lEV formation. The plasma membrane consists of an inner and outer leaflet, and lipids distribute asymmetrically between them [69]. At resting conditions, aminophospholipids (including PS) and phosphatidylethanolamine are in the inner leaflet of the plasma membrane, while phosphatidylcholine and sphingomyelin are in the outer leaflet. This lipid distribution is kept in place by the action of flippase enzymes, flippase works at resting state by back-transportation of PS or phosphatidylethanolamine from the outer leaflet to the inner leaflet of the plasma membrane in ATP-dependent manner. When cells are activated or undergoing apoptosis, floppases, an ATP-dependent protein, act by translocating inner leaflet lipids to the outer leaflet, thus resulting in disruption of the membrane asymmetry. Moreover, Ca^2+^ influx inhibits the action of flippase, allowing PS and phosphatidylethanolamine to externalize rapidly by the action of floppase. Ca^2+^ influx also activates the non-specific lipid transporter “scramblase”, which works by moving phospholipid down their concentration gradient [69,70]. Meanwhile, Ca^2+^-dependent calpain is activated to proteolyze the cytoskeleton as discussed earlier and leads to outward membrane blebbing and lEV shedding. The externalization of PS and associated membrane alterations appear to be vital mediators of lEV shedding from the plasma membrane, although generation of lEV populations without PS exposure measured by annexin V binding was reported [71]. Overall, the exact mechanism of lEV formation is not well known, and it appears to differ from one cell type to another, but in general, cytoskeletal disruption and PS externalization seem to be involved in lEV formation. Understanding the molecular mechanisms that lead to lEV formation and release allows the development of therapeutics that target formation and release processes.

### 5.2. Content of lEVs

With time, lEVs (MPs) have been shown to contain biological material including lipids, proteins and RNAs (Figure 3C). Proteomic analysis revealed the protein content of lEVs and provided information required for detecting lEVs from different cellular origins in order to investigate their role in physiology and disease. Banfi et al. [72] investigated the protein content of endothelial lEVs following stimulation with TNF-α and identified many cytoskeleton proteins, membrane proteins and other proteins involved in cell signaling [72]. Additionally, Palmisano et al. [73] quantified and evaluated membrane-protein content of lEVs derived from cytokine-stimulated pancreatic β-cells and found 401 proteins, many of them were involved in tumor necrosis factor signaling pathway, which regulates inflammatory and immune response as indicated by network analysis [73]. Moreover, it appears that the protein packaging is differential based on the type of the stimulus exerted on the cell. A proteomic analysis of plasma lEVs derived from patients with newly diagnosed type-2 diabetes and healthy participants showed differences in the protein content between them. For instance, 46 proteins were shown to be deferentially expressed between diabetic patients’ lEVs and control subjects’ lEVs, with existence of new proteins in diabetic patients’ lEVs only. Additionally, pathway analysis indicated that proteins detected in lEVs from diabetic patients were involved in cell adhesion, platelet activation and inflammation, which suggest their association with pathological manifestations of diabetes [74]. There is belief that the protein content of lEVs mirror that of the mother cell. In a work done recently by Fan et al. [75], protein disulfide isomerase (PDI) was found to be increased in the plasma of metabolic syndrome patients as well as in the isolated lEVs from these patients [75].

Lipid composition of lEVs seems to be like that of the plasma membrane of secreting cells since MPs are released directly from the plasma membrane. A phospholipidomic study has found that red blood cells and their derived lEVs have similar lipid profiles except for polyunsaturated glycerophosphoserines, which were enriched in lEVs [76]. This might provide more rigidity to the lEV membrane, allowing them to be good carriers of biological materials. Moreover, lEVs harbor PS at their surface membrane, which results in high procoagulant capacity of lEVs through providing sites for coagulation factors to bind and initiate coagulation cascades. This coagulation capacity was recognized very early in 1967 when lEVs were believed to be platelet dust and their function was not yet revealed [77]. Sinauridze et al. [78] showed that a platelet-derived lEV had similar pro-coagulant activity as an activated platelet despite the smaller surface area of lEVs, which will make them more pro-coagulant than activated platelets [78]. This procoagulant nature is involved in physiological and pathophysiological effects exerted by lEVs. Additionally, it was reported that lEVs contain genetic material, specifically small RNA of less than 200 nucleotides such as microRNA and that they can deliver this content to recipient cells [79].

### 5.3. Mechanisms of lEVs (MPs) Interaction with Target Cells

Cumulative evidence has shown that lEVs are able to release their cargo content by different means of interaction with target cells and considered as vectors of biological messages [80], both in health and disease [81]. Due to their presence in blood circulation, they can interact and communicate with surrounding vascular cells including endothelial cells. After lEVs are released from the cell membrane, they can directly interact with target cells via ligand/receptor interaction to generate cell signaling. Another possible mechanism of interaction is transferring proteins such as membrane receptors and adhesion molecules from lEVs to the target cell surface. Direct fusion and release of all lEV’s content inside the target cell or endocytosis were also proposed as means of lEV interaction with target cells. However, the exact mechanisms of interaction are not yet well known. Altogether, lEVs contain biological messages and can convey them to surrounding cells through different mechanisms leading to alterations in cellular activity of target cells.

## 6. lEVs (MPs) and Endothelial Dysfunction

lEVs (MPs) have emerged as useful biomarkers and predictors of endothelial dysfunction, which is known to be associated with metabolic abnormalities including obesity, diabetes and metabolic syndrome. lEVs levels are altered in obesity. For example, CD51+ endothelial lEVs were shown to be significantly increased in patients with hypertension and obesity compared to patients with hypertension only and healthy controls [82]. Additionally, CD144+ endothelial lEVs were significantly increased in overweight and obese children [83]. Esposito et al. [84] found CD31+/CD42b− endothelial lEVs and CD31+/CD42b+ platelet lEVs to be significantly elevated in obese women with concomitant reduction in endothelium-dependent flow mediated vasodilation. The same study found a significant positive correlation between endothelial and platelet lEVs, and waist-to-hip ratio as a sign of central obesity. Endothelial lEVs were independent predictors of endothelial dysfunction, as shown by a reduction in endothelium-dependent flow-mediated vasodilation in obese women [84]. Therefore, endothelial lEVs were suggested as useful biomarkers for early detection of endothelial dysfunction in obesity.

Studies also investigated the levels of EVs in diabetes. In 2004, two studies conducted by two separate groups evaluated the effect of endothelial lEVs [85], and circulating lEVs from diabetic patients and in vitro*-*generated T-lymphocyte lEVs [86], on endothelial cell function and concluded a deleterious role of lEVs in endothelial dysfunction. Feng et al. [87] have found similar observations to the study by Esposito et al., but in diabetic patients. CD31+/CD42− and CD51+ endothelial lEVs, annexin V+ lEVs, platelet lEVs, and lymphocyte-derived lEVs, were significantly increased in diabetic patients compared to healthy controls [87]. Correlation and regression analysis indicated significant positive correlation between endothelial lEVs and HbA1c level, and that endothelial lEVs are independent predictors of endothelial dysfunction (reduced flow-mediated dilation of brachial artery and elevated brachial ankle pulse wave velocity) in diabetic patients, respectively [87]. Feng et al. [88] and Ishida et al. [89] showed significantly elevated levels of circulating lEVs from endothelial, leukocyte and platelet origins in STZ-induced diabetic rats, compared to non-diabetic rats [88,89].

EVs were shown to contribute to the pathogenesis of endothelial dysfunction in diabetes [90,91,92,93]. For example, endothelial lEVs predominantly express miR-126, which can induce endothelial cell repair after vascular injury by increasing endothelial cell migration and proliferation, which are important steps in angiogenesis. In diabetic patients, significantly decreased expression of miR-126 altered its protective role of the endothelium [90]. Moreover, sirtuin 6 mRNA, a member of nicotinamide adenine dinucleotide-dependent histone deacetylases family that has pathophysiological roles in apoptosis and DNA damage and repair, when incorporated in lEVs results in enhancing endothelial cell protection. However, its mRNA expression was decreased in lEVs isolated from high glucose-treated HUVECs and endothelial lEVs isolated from diabetic patients, which resulted in endothelial dysfunction [94]. Notably, Western blot analysis did not reveal the expression of sirtuin 6 in endothelial lEVs isolated from diabetic patients or healthy controls and lEVs isolated from high glucose treated HUVECs or non-treated HUVECs. This might indicate that endothelial protection is a result of serum sirtuin 6, which was also affected by high glucose levels, and not from sirtuin 6 incorporated into MPs. Giannella et al. [91] showed that miR-126-3p encapsulated in lEVs was significantly reduced as individuals progressed to prediabetes and diabetes. Moreover, miR-126-3p content inversely correlated with CD62E+ lEVs and plasma glucose levels [91]. Altered protein composition of lEVs was confirmed by the proteomic analysis of cultured HUVECs induced with high glucose [92].

Metabolic syndrome is a group of abnormalities including elevated triglyceride level, reduced high-density lipoproteins (HDL), elevated blood pressure, hyperglycemia and central obesity that increases the individual’s risk of developing cardiovascular diseases and type-2 diabetes [95]. In metabolic syndrome patients, endothelial [96], procoagulant, platelet, and erythrocyte lEVs were significantly elevated compared to healthy individuals [97]. Agouni et al. [97] have found that lEVs derived from metabolic syndrome patients were able to induce endothelial dysfunction both in vitro and in vivo. Treatment of endothelial cells with metabolic syndrome lEVs decreased NO production and increased the phosphorylation of eNOS at the inhibitory site (Thr495), while injection of these lEVs into mice attenuated the relaxation of endothelium in response to acetylcholine in aortas [97].

Collectively, the link between ER stress activation and endothelial dysfunction in insulin resistance states such as hyperglycemia and obesity, was proven in vitro and in vivo as well as in humans. In addition, in the same pathological conditions, lEVs levels were shown to be altered and contribute to endothelial dysfunction (Table 2, Table 3 and Table 4).

## 7. Crosstalk between ER Stress and lEVs (MPs) in Endothelial Dysfunction

The relationship between ER stress and lEVs (MPs) in the pathogenesis of endothelial dysfunction is not fully studied; however, some initial studies identified a crosstalk and a two-way relationship between ER stress activation and lEVs in the development of endothelial dysfunction. Safiedeen et al. [11] have shown a role of ER stress activation in endothelial dysfunction induced by lEVs. In vitro treatment of human aortic endothelial cells (HAoECs) with lEVs derived from metabolic syndrome patients or generated from apoptotic T-lymphocytes, activated all UPR three arms: PERK, IRE-1α, and ATF-6, reduced NO bioavailability in vitro, and impaired endothelium-dependent vasodilation in vivo in mice, all actions that were reversed by the presence of TUDCA, an ER stress inhibitor [11]. These results show the involvement of ER stress in endothelial dysfunction caused by lEVs derived from metabolic syndrome patients and apoptotic T-lymphocytes.

In addition to the role of ER stress in inducing lEV-mediated endothelial cell dysfunction, Jia et al. [99] isolated ER stress-dependent lEVs from smooth muscle cells and human aortic endothelial cells, both induced by mechanical stretch. It is important to note that both cell types are closely connected, and the function of vascular smooth muscle cells is directly affected by the endothelial cells’ health state. Treatment with PBA, a chemical chaperone that prevents ER stress, before the activation of cells with mechanical stretch not only decreased lEV production from the two cell lines but also decreased pathological effects mediated by these vesicles [99]. More recently, lEVs generated from cultured endothelial cells subjected to pharmacologically (thapsigargin)-induced ER stress activated ER stress in endothelial cells in a vicious circle suggesting a two-way relationship between ER stress and release of lEVs [12]. Furthermore, ER stress-generated lEVs impaired the angiogenic capacity of HUVECs in a mechanism independent from cells survival and autophagy [12].

## 8. Conclusions

Multiple clinical uses for lEVs were explored in the literature. One of these is to assess and follow-up the efficacy of treatments by monitoring changes in the levels of lEVs [100]. Certain pharmacotherapies, such as statins and acetylsalicylic acid, were reported to reduce circulating levels of lEVs [101,102,103,104]. In addition, because of their ability to carry biological content and transfer it to target cells, lEVs can also be used as therapeutic tools [80,105]. Certain types of lEVs were reported to enhance angiogenesis and improve revascularization in animal models of ischemia [106,107]. lEVs can also be considered as effective carriers and non-toxic targeting tools for gene therapy because of their capacity to harbor and protect genetic material such as microRNA and deliver it to target cells [80,100,105,108]. The pro-coagulant and pro-thrombotic actions of lEVs were also tested in the management of certain bleeding disorders such as thrombocytopenia [109,110]. Monitoring the changes in the expression levels of lEVs from various cellular origins is another key promising application for lEVs that can represent effective biomarkers for disease diagnosis and prognosis. For instance, several studies compared the patterns of shedding of lEVs between apoptotic and activated endothelial cells to generate scores to assess heart failure and predict cardiovascular risks [111,112,113].

The production of lEVs in pathological conditions, such as obesity and diabetes, which are characterized by the activation of ER stress, were shown to initiate endothelial dysfunction in a two-way relationship. Therefore, lEVs can be targeted by controlling their release from the cells, inhibiting their cellular uptake by the recipient cell or modulating their cargo content. Controlling the release of EVs through different drugs including antidiabetic agents was investigated and interestingly, these agents were shown to modulate the levels of EVs in diabetic patients [114,115,116]. Furthermore, improving our understanding of the deleterious messages carried out by EVs in pathological conditions characterized by sustained activation of ER stress is critical to identify novel specific therapeutic or preventative targets against endothelial dysfunction, the first step in atherogenesis and cardiovascular complications.

## Figures and Tables

**Table 1:** Factors produced by the endothelium that contribute to vascular homeostasis.

Relaxing Factors	Contracting Factors	Contracting Factors	Antithrombotic Factors	Growth Factors	Inflammatory Mediators
Nitric oxide (NO) Prostacyclin (PGI2) Endothelium-derived hyperpolarizing factor (EDHF)	Endothelin-1 (ET) Constricting prostaglandins: Thromboxane (TXA2) and Prostaglandin H2 (PGH2) Arachidonic acid metabolite: 20-hydroxyeicosatetraenoic acid (20-HETE)	Von Willebrand factor (vWF) TXA2 Platelet activating factor Plasminogen activator inhibitor Annexin II	NO and PGI2 Antithrombin Heparin Tissue plasminogen activator Thrombomodulin Annexin V Proteoglycans Protein C Protein S	Insulin like growth factor Transforming growth factor Colony stimulating factor	Interleukins (IL)- 1,6,8 Leukotrienes Major histocompatibility complex (MHC) II

**Table 2:** Summary of the main studies of altered lEV (MP) levels and their associated effects on obesity in human, in vitro and animal models.

Origins of MPs and Markers Used for Detection	Human, Cell and Animal Models	Key Observations	References
OBESITY			
Endothelial lEVs (CD51+, CD144+, and CD31+/CD42b−)	Obese patients with hypertension (HTN)	CD51+ lEVs were significantly elevated in obese patients with HTN than HTN only patients. CD31+/CD42b− lEVs were significantly elevated in obese and non-obese hypertensive patients compared to healthy control.	Hu et al. [82]
Endothelial lEVs (CD144+ and CD146+)	Overweight and obese children	CD144 + lEVs and left ventricular mass index were significantly higher in overweight and obese children with elevated blood pressure levels and insulin resistance compared to control.	Gündüz et al. [83]
Endothelial lEVs (CD31+/CD42b−) and platelet lEVs (CD31+/CD42b+)	Obese women	Obese women with signs of insulin resistance had significant elevation in endothelial and platelet lEVs levels and significant reduction in endothelium-dependent flow-mediated dilation (FMD). Correlation analysis indicated the following: ▪ Waist-to-hip ratio, but not Body Mass Index (BMI), significantly and positively correlated with lEV levels. ▪ MP levels negatively correlated with FMD. Multivariate analysis indicated that endothelial lEVs are predictors of endothelial dysfunction in this population.	Esposito et al. [84]

**Table 3:** Summary of the main studies of altered lEV (MP) levels and their associated effects on diabetes in human, in vitro and animal models.

Origins of MPs and Markers Used for Detection	Human, Cell and Animal Models	Key Observations	References
DIABETES			
Platelet, endothelial and leukocyte lEVs	STZ-induced diabetic rats	Plasma lEVs were significantly elevated in diabetic rats, with greater PS exposure. Diabetic lEV was able to adhere to microvascular endothelium and enhanced leukocyte adhesion through binding to PS on their surface.	Feng et al. [88]
Endothelial lEVs	Cultured HUVECs induced with high glucose for 24 h	Endothelial lEV level was significantly higher and possess significant procoagulant activity in HUVECs treated with high glucose compared to non-treated or osmotic controlled HUVECs. Proteomic analysis revealed altered protein composition of high glucose-derived endothelial lEVs. Endothelial lEVs impaired endothelium mediated vasorelaxation in arteries.	Burger et al. [92]
Endothelial lEVs (CD31+/CD42−, CD62+/CD42−, CD42+)	Cultured HUVECs induced with high glucose (33 mM) Diabetic patients	Endothelial lEVs (apoptotic: CD31+/CD42− and activated CD62+/CD42−) were significantly elevated in the plasma of diabetic patients. Apoptotic CD31+ endothelial lEVs were significantly elevated in HUVECs induced with high glucose. Decreased serum Sirtuin 6 and mRNA expression of Sirtuin 6 in endothelial lEVs resulted in endothelial dysfunction (impaired angiogenesis and decreased NO concentration).	Jing et al. [94]
Endothelial lEVs (CD62E+, Annexin V+/CD62E+, Annexin V+)	Prediabetic and diabetic patients	Activated endothelial lEVs (CD62E+) were significantly elevated in prediabetic and diabetic patients. Annexin V+/CD62E+ and Annexin V+ lEVs were significantly elevated in type-2 diabetic patients compared to control and prediabetics. There is significant negative association between CD62E+ level and glucose levels. miR-126-3p encapsulated in lEVs is significantly reduced as individual progress to prediabetes and diabetes. miR-126-3p content inversely correlated with CD62E+ lEV and plasma glucose levels.	Giannella et al. [91]
Platelet lEVs (P-selectin (CD62P+) and CD61+)	STZ-induced male Wistar rats	Circulating lEVs (mainly of platelet origin) were significantly elevated in diabetic rats. Incubation of control carotid artery rings with MPs from STZ-induced diabetic rats impaired acetylcholine-induced endothelium-dependent relaxation. lEVs induced endothelial dysfunction through decreasing eNOS expression and increasing of caveolin-1 (CAV-1) expression.	Ishida et al. [89]
AnnexinV+ lEVs, endothelial lEVs (CD31+/CD42− and CD51+)	Patients with type-2 diabetes	AnnexinV+, platelet, leukocyte, endothelial (CD31+/CD42− and CD51+) lEVs were all significantly elevated in patients with diabetes compared to healthy controls. HbA1c is positively and significantly correlated with endothelial lEVs (CD31+/CD42−). Signs of endothelial dysfunction (decreased flow mediated dilation and increased pulse wave velocity) in diabetic patients were significantly correlated with high endothelial lEV levels.	Feng et al. [87]
Lymphocyte lEVs and circulating lEVs	Lymphocyte lEVs derived from: Human lymphoid CEM T cell line induced with actinomycin D or phytohemagglutinin Patients with type-2 diabetes Circulating lEVs derived from type-2 diabetes or HIV patients	lEVs from activated human lymphoid CEM T cell line reduced endothelium-dependent relaxation and sheer stress-induced dilation. They decreased expression of eNOS in HUVECs and mouse aortic rings. They induced CAV-1 expression in HUVECs (lEVs themselves did not express CAV-1). Other lEVs populations reduced expression of eNOS in vitro.	Martin et al. [86]
Platelet, leukocytes and monocytes lEVs	Patients with diabetes and obese patients	In diabetic patients, total platelet lEVs, fibrinogen-positive platelet lEVs, tissue factor-positive platelet lEVs, P-selectin positive platelet lEVs were significantly elevated compared to non-diabetic participants. Other lEVs populations were not altered in diabetic patients. lEV derived from obese participants did not affect lEV levels.	Zhang et al. [98]

**Table 4:** Summary of the main studies of altered lEV (MP) levels and their associated effects on metabolic syndrome in human, in vitro and animal models.

Origins of MPs and Markers Used for Detection	Humans, Cell and Animal Models	Key Observations	References
METABOLIC SYNDROME			
Procoagulant lEVs (annexin V+), platelet (CD41+), endothelial (CD146+), erythrocyte (CD235a+) and leukocyte (CD45+) lEVs	Metabolic syndrome patients	The level of procoagulant, platelet, endothelial, erythrocyte and leukocyte lEVs were significantly higher in metabolic syndrome patients compared to non-metabolic syndrome individuals. In vitro treatment with metabolic syndrome lEVs: ▪ activated ER stress (increase p-eIF2*α* expression and nuclear translocation of ATF-6) ▪ reduced NO levels In vivo treatment with metabolic syndrome lEVs significantly reduced endothelium depended relaxation. ER stress and endothelial dysfunction were prevented with TUDCA.	Safiedeen et al. [11]
Circulating lEVs, procoagulant (annexin V+), platelet (CD41+), endothelial (CD146+), and erythrocyte (CD235a+) lEVs	Metabolic syndrome patients	Total, procoagulant, platelet, endothelial, and erythrocyte lEVs were significantly elevated in metabolic syndrome patients compared to healthy controls. Most lEVs were from platelet origin. In vitro, metabolic syndrome lEVs significantly reduced the NO in endothelial cells and increased expression of p-eNOS at the inhibitory site Thr495 but did not affecting CAV-1 expression. In vivo injection of metabolic syndrome lEVs decreased eNOS expression and impaired endothelial-dependent relaxation in mice aorta.	Agouni et al. [97]
Platelet (CD31+), endothelial (CD31+, CD62E+ and CD51+) lEVs	Metabolic syndrome patients	Metabolic syndrome patients have significantly higher levels of apoptotic endothelial lEVs (CD31+) compared to healthy participants.	Arteaga et al. [96]
